# Copy Number Variation in Thai Population

**DOI:** 10.1371/journal.pone.0104355

**Published:** 2014-08-13

**Authors:** Bhoom Suktitipat, Chaiwat Naktang, Wuttichai Mhuantong, Thitima Tularak, Paramita Artiwet, Ekawat Pasomsap, Wallaya Jongjaroenprasert, Suthat Fuchareon, Surakameth Mahasirimongkol, Wasan Chantratita, Boonsit Yimwadsana, Varodom Charoensawan, Natini Jinawath

**Affiliations:** 1 Department of Biochemistry, Faculty of Medicine Siriraj Hospital, Mahidol University, Bangkok, Thailand; 2 Department of Biochemistry, Faculty of Science, Mahidol University, Bangkok, Thailand; 3 Enzyme Technology Laboratory, Bioresources Technology Unit, National Center for Genetic Engineering and Biotechnology (BIOTEC), Pathumthani, Thailand; 4 Faculty of Information and Communication Technology, Mahidol University, Nakhon Prathom, Thailand; 5 Division of Virology and Molecular microbiology, Department of Pathology, Faculty of Medicine Ramathibodi Hospital, Mahidol University, Bangkok, Thailand; 6 Endocrine and Metabolism Unit, Department of Medicine, Faculty of Medicine Ramathibodi Hospital, Mahidol University, Bangkok, Thailand; 7 Thalassemia Research Center, Institute of Molecular Biosciences, Mahidol University, Salaya, Nakhon Prathom, Thailand; 8 Medical Genetics Section, National Institute of Health, Department of Medical Sciences, Ministry of Public Health, Nonthaburi, Thailand; 9 Research Center, Faculty of Medicine Ramathibodi Hospital, Mahidol University, Bangkok, Thailand; 10 Integrative Computation BioScience Center (ICBS), Mahidol University, Nakhon Prathom, Thailand; Seoul National University College of Medicine, Republic Of Korea

## Abstract

Copy number variation (CNV) is a major genetic polymorphism contributing to genetic diversity and human evolution. Clinical application of CNVs for diagnostic purposes largely depends on sufficient population CNV data for accurate interpretation. CNVs from general population in currently available databases help classify CNVs of uncertain clinical significance, and benign CNVs. Earlier studies of CNV distribution in several populations worldwide showed that a significant fraction of CNVs are population specific. In this study, we characterized and analyzed CNVs in 3,017 unrelated Thai individuals genotyped with the Illumina Human610, Illumina HumanOmniexpress, or Illumina HapMap550v3 platform. We employed hidden Markov model and circular binary segmentation methods to identify CNVs, extracted 23,458 CNVs consistently identified by both algorithms, and cataloged these high confident CNVs into our publicly available *Thai CNV* database. Analysis of CNVs in the Thai population identified a median of eight autosomal CNVs per individual. Most CNVs (96.73%) did not overlap with any known chromosomal imbalance syndromes documented in the DECIPHER database. When compared with CNVs in the 11 HapMap3 populations, CNVs found in the Thai population shared several characteristics with CNVs characterized in HapMap3. Common CNVs in Thais had similar frequencies to those in the HapMap3 populations, and all high frequency CNVs (>20%) found in Thai individuals could also be identified in HapMap3. The majorities of CNVs discovered in the Thai population, however, were of low frequency, or uniquely identified in Thais. When performing hierarchical clustering using CNV frequencies, the CNV data were clustered into Africans, Europeans, and Asians, in line with the clustering performed with single nucleotide polymorphism (SNP) data. As CNV data are specific to origin of population, our population-specific reference database will serve as a valuable addition to the existing resources for the investigation of clinical significance of CNVs in Thais and related ethnicities.

## Introduction

Copy Number Variation (CNV) is one of the major genetic variations observed among genomes of individuals. CNVs constitute more total nucleotides than Single Nucleotide Polymorphisms (SNP), accounting for almost 12% of the human genome, and are of important in terms of genetic diversity as well as human evolution [1]. At present, several conditions with genetic etiologies, such as autism spectrum disorder, developmental delay, and non-syndromic multiple congenital anomalies, are well documented to have CNVs among the causative variants [2]. For this reason, array-based technology, which is commonly used for CNV identification, has been recommended as a first-tier diagnostic tool for these particular disorders [3]. To make an accurate clinical interpretation of CNVs, both databases containing reference CNVs from genetic disease patients and normal controls are required. Large databases consisting of CNVs and clinical information of patients with chromosomal disorders such as DECIPHER [4] and the International Collaboration for Clinical Genomics (ICCG; http://www.iccg.org/) are actively curated by working groups. However, most patients are of European descent due to the availability and easy accessibility of clinical CNV testing in North America and Europe. Apart from these, there are currently a few other large public CNV databases containing CNV information of control subjects from certain ethnic groups, such as Caucasian, African-American, and Asian American [5], [6]. These general population databases greatly help with clinical interpretation of CNVs, which can be divided into three main categories: pathogenic, uncertain clinical significance, or benign [7]. Recently, publications focusing on CNVs of specific ethnicities such as Koreans [8], Europeans [9], and Chinese [10] emphasize the fact that there are significant amount of population-specific CNVs. So far the number of Thai individuals represented in the existing databases for CNV in general population is very limited [11], and thus they are by no means the ideal references for CNV interpretation in Thais. The International Haplotype Map Project phase III (HapMap3) has made publicly accessible SNP genotyping and CNV data of more than a thousand subjects from 11 different ethnic groups, e.g. European, African, and East Asian ancestries [12]. HapMap3 dataset provides an opportunity to compare genetic variations across populations. Hence, CNVs in a larger sample of Thai individuals can be characterized and distinguished from those of East Asian and other populations.

In this study, we combined the genomics data generated from multiple genome-wide association studies (GWAS) consisting of 3,017 unrelated Thai subjects with no undiagnosed genetic disorders. We carried out CNV discovery from these dataset using the two commonly used CNV calling algorithms, PennCNV [13] and CNV Workshop [14], to identify the most accurate set of CNVs, and put together the first large reference CNV database for Thais. Furthermore, we performed population Copy Number Variation Region (CNVR) frequency comparison between Thais and 11 HapMap3 populations, and identified unique CNVRs in Thais as well as CNVs overlapping with genes associated with Thai population. Genetic similarity between each population was also explored using hierarchical clustering analysis (HCA) based on the CNV frequencies. The Thai CNV database should contribute to a more accurate clinical interpretation of CNVs in Thai patients and serve as the starting point for future population genetics and genetic epidemiology studies.

## Materials and Methods

### Study populations

The study population were compiled from previously published genome-wide association studies (GWAS) in Thai individuals [15], [16], [17], [18], [19], which were generated under collaborations between the Ministry of Public Health, Thailand, Thailand Center of Excellence for Life Sciences (TCELS), and the RIKEN Center for Genomic Medicine (CGM), Japan (Table 1), and CNV data of 11 different ethnic groups publicly available through the HapMap3 project (Table S1 in File S1) [14]. This study was approved by a Committee on Human Rights Related to Research Involving Human Subjects, Faculty of Medicine Ramathibodi Hospital, Mahidol University.

**Table 1:** GWAS studies containing the genomics data of 3,017 Thai individuals after exclusion of low quality samples.

Reference	Type of SNP array	Number of subjects	Total	Excluded (%)
Jongjaroenprasert et al, 2012	Illumina Human610-quad	289	330	12.424
Mahasirimongkol et al, 2012	Illumina Human610-quad	463	484	4.339
Wattanapokayakit et al (unpublished data)	Illumina HumanOmniExpress-12	517	685	24.526
Chantarangsu et al, 2011	Illumina HumanHap550-Duo v3	56	165	66.061
Chantarangsu et al, 2011	Illumina Human610-quad	167	210	20.476
Mahasirimongkol et al, 2012	Illumina Human610-quad	856	868	1.382
Nuinoon et al, 2010	Illumina Human610-quad	669	685	2.336
Total		3,017	3,427	11.964

### CNV discovery in Thai population

Genotype data in the Thai population were generated using Illumina Human610-Quad, or Illumina HapMap 550v3, or Illumina HumanOmniexpress genotyping platform (Illumina, San Diego, CA, USA) (Table 1). Signal Intensity data in 3,427 Thai individuals were obtained. Individual samples with SD of log-R ratio >0.3, with SNP call rate of <98%, or with self-reported/genotype-derived sex inconsistency were excluded leaving a total of 3,017 Thais prior to subsequent analyses. Intensity data of SNPs that were not in Hardy-Weinberg equilibrium (HWE) using a threshold of 10^−5^ were excluded prior to CNV prediction as previously described [17].

Two CNV prediction algorithms, Hidden Markov Model (HMM) and Circular Binary Segmentation (CBS), were used to call CNVs from signal intensity in the Thai population. CNV discovery using an HMM-based algorithm was performed with PennCNV software version 2011Jun16 [13]. Briefly, the intensity data of A and B alleles from raw files were extracted, normalized, and transformed into Log R Ratio (LRR) and B Allele Frequency (BAF) using GenomeStudio software (Illumina, San Diego, CA, USA). Population frequency of B allele file (pfb) for Thai population was estimated and used together with HMM model file provided by PennCNV software. LRR and BAF at each probe location were then used to predict one of the four possible states of CNV: homozygous deletion, heterozygous deletion, normal copy number, and at least one copy duplication.

A CBS-based algorithm was implemented in CNV Workshop [14]. For CBS, LRR data were used to identify a segment in the genome that displays a change in signal intensity. Mean LRR and distribution of BAF were then used to predict how likely each segment of the genome is a copy number variant. CNVs were then called using default parameters. The CNV statistics illustrating the characteristics of HMM and CBS were summarized in (Table S2 in File S1).

CNVs in HapMap3 populations were downloaded from HapMap project website, http://HapMap.ncbi.nlm.nih.gov/downloads/cnv_data/hm3_cnv_submission.txt on March 12, 2014. Family information and population origin of the samples were obtained from Coriell Cell Repositories (http://ccr.coriell.org/) using an in-house python script. The same quality control criteria used to filter CNVs in Thai populations were applied to HapMap3 data. After excluding offspring, there were 79,517 CNVs in 1,038 individuals left for subsequent analysis.

### Quality control of CNV data

**Figure 1:**
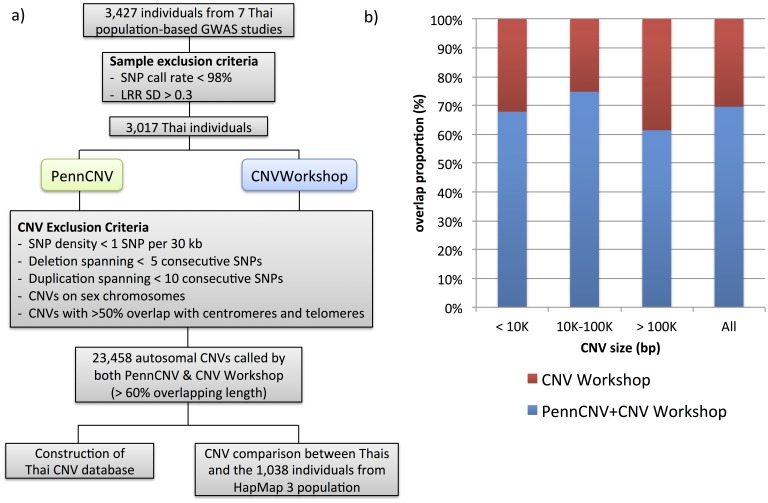
CNV discovery in the Thai population. a) Diagram showing Thai CNV discovery workflow; b) % overlap proportion of CNVs identified by both CNV Workshop and PennCNV based on CNV size (bp). The regions shaded in red correspond to CNVs exclusively discovered by CNV Workshop, while regions shaded in blue represent those jointly discovered by CNV Workshop and PennCNV.

The CNVs predicted by HMM algorithm were verified with the results from CBS algorithm. For each subject, CNVs called by both HMM and CBS algorithms, with at least 60% overlapping length were considered replicable. These overlapped regions were used as the start and end of CNVs in subsequent analyses. To minimize false positive results, we only included CNVs with at least 30 kb/SNP density, at least 5 SNPs (>5 kb) for deletion CNVs, at least 10 SNPs (>10 kb) for duplication CNVs. CNVs overlapped more than 50% with centromeric and telomeric regions, and CNVs on sex chromosome were excluded (Figure 1a). Individuals predicted to have more than 100 CNVs, most likely from an error from genotyping array, were also excluded [14].

### CNV distribution among population

Pairwise comparison of the frequencies of CNVs between Thais versus each of the 11 HapMap3 populations was performed using the test of association function implemented in PLINK (http://pngu.mgh.harvard.edu/~purcell/plink/cnv.shtml) [20]. The empirical statistical significant level was calculated using 5,000 permutations. CNVs with statistically significantly different frequency were defined as any CNV with empirical p-value <0.0002 (1 in 5000 chance). CNV loci encompassing genes were exclusively chosen, and their frequencies in each population were calculated. To identify CNVs with the greatest frequency difference between the Thai and each HapMap3 population, 20 genes (p<0.0002) comprising of the top 10 genes with higher frequencies, and the bottom 10 genes with lower frequencies in the Thai population were selected for each of the pairs. These CNV frequencies across all populations were subsequently used to performed hierarchical clustering analysis. CNV frequencies were scaled and centered to have a mean of 0 and a variance of 1. Hierarchical clustering using Euclidean distance with Ward clustering method was performed on the scaled frequencies using *pheatmap* package in R version 3.0.1 [21].

### Copy number variable region

In this study, we applied a widely used term Copy Number Variable Region (CNVR) to represent a discrete region in the genome that overlaps with CNVs. After combining CNV data from the Thai and HapMap3 populations, CNVRs were defined by merging overlapping CNVs into a discrete region using *GenomicRanges* packages in R. The frequencies of CNVRs in each population were calculated by counting the number of individuals whose CNV(s) fell within each predefined CNVR divided by the total number of people in each population. CNVRs with at least 5% frequency in Thais were defined here as common CNVRs. CVNRs overlapping with gene regions were identified using *GenomicRanges* package in R using the gene list based on hg18 data downloaded from PLINK software resource page (http://pngu.mgh.harvard.edu/~purcell/plink/dist/glist-hg18). To identify the degree of match between the Thai and HapMap3 CNVRs, CNVRs were created separately using either CNVs from Thais or the HapMap3 populations. Only CNVs available in more than one individual were included in this analysis.

## Results

### Characteristics of CNV in the Thai population

After excluding the samples with poor quality data including low SNP call rates and high LRR SDs, there were 3,017 individuals left for subsequent analyses (Table 1 and Figure 1a). Among the 42,290 CNVs identified by CNV Workshop, 29,436 CNVs (70%) were consistently predicted by PennCNV (Figure 1b). We extracted the most confident CNV dataset possible by excluding CNVs represented by sparse probe coverage (<1 SNP per 30 kb), small deletion (<5 consecutive SNPs) or small duplication (<10 consecutive SNPs). CNVs overlapping with centromeric and telomeric regions as well as sex chromosomes were also excluded due to a high false positive CNV prediction rates. After these filtering processes, there were 23,458 CNVs that passed these criteria, and thus kept for subsequent analyses. The CNV size ranged from 5 kb to 4.28 Mb, with a median of 59,804 bp (Table 2). Up to 23 CNVs were identified in each individual, with the median number of eight CNVs per genome. Overall, we observed more deletion CNVs (79%) than duplication CNVs (21%). The median size of CNVs was 40,811 bp for deletion and 122,757 bp for duplication CNVs. The higher amount of deletion CNVs as compared to duplication CNVs may reflect the power of current CNV calling algorithms to preferably detect smaller deletions. The largest CNV identified was a duplication of 4.28 Mb spanning chromosome 12q14 to 12q15. Although this CNV overlapped with a known 12q14 microdeletion syndrome documented in the DECIPHER database v7.0 (https://decipher.sanger.ac.uk/syndromes) [4], there was no reported clinical significance of duplication of the same region. Among the 23,458 CNVs identified in Thai population, 766 CNVs (3.27%) were overlapped with known chromosomal imbalance syndromes curated in the DECIPHER database with reference to matched CNV type.

**Table 2:** Thai CNV and CNVR characteristics.

	Thai CNVs	Thai CNVRs
Total count	23,458	1,014
Duplication CNVs	4,879	165
Deletion CNVs	18,579	538
Complex CNVs		311
Median (mean) number per genome	8 (7.77)	7 (7.35)
Median size (range) (kb)	59.80 (5.0–4275.08)	95.06 (5.18–4275.08)
Median size of duplications	122.76 (100.45–4275.08)	137.34 (14.67–1491.4)
Median size of deletions	40.81 (5.0–3893.87)	37.5 (5.18–2144.0)
Genome coverage		261.77 Mb (8.72%)

### Thai CNVs versus HapMap3

**Figure 2:**
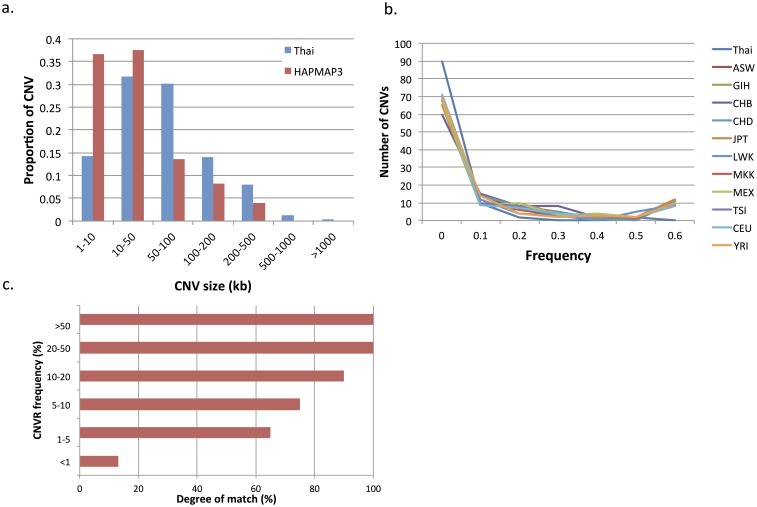
CNV and CNVR comparison between the Thai and eleven HapMap3 populations. a) Size distribution of the Thai CNVs and HapMap3 CNVs; b) Allele frequency spectrum of CNVs with frequency of at least 1% across the Thai and HapMap3 CNVRs; c) Degree of match between the Thai CNVRs and HapMap3 CNVRs with reference to allele frequency.

The overall frequency and size distribution of CNVs in the Thai population was relatively similar to the frequency distributions in 11 other HapMap3 populations (Figure 2a and 2b). Comparing the CNV sizes, we observed that HapMap3 had higher amount of small CNVs (<50 Kb) than those in the Thai population (Figure 2a). When CNVs were combined into discrete regions within the HapMap3 populations, after excluding CNVs found only in a single individual, there were 506 discrete CNVRs. Considering only CNVRs created from the Thai CNVs, 822 (81.14%) out of 1,014 CNVRs did not overlap with any CNVRs in HapMap3, while there were 192 CNVRs that were common to both Thais and HapMap3. The median size of these shared CNVRs was 174.1 kb, with a mean allele frequency of 2.65%. The median size of CNVRs found only in the Thai population was 83.8 kb, with a mean allele frequency of 0.26%. The CNVRs shared between Thai and HapMap3 populations were both statistically significantly larger and more common than the Thai-specific CNVRs (p-value <0.001). All common CNVRs with frequency above 20% could be found in both Thais and HapMap3. As CNVRs became less frequent, the proportion that these CNVRs exist in both populations became lower (Figure 2c).

After combining CNV data in the Thai population together with those from HapMap3, 2,560 CNVRs were defined. Most CNVRs (60%) were found only in one individual. Common CNVRs with at least 5% frequencies in the Thai population were summarized and contrasted with the HapMap3 populations in Table 3. The most common CNVR (hg18 location chr4: 69,045,672–69,258,302) in Thais was found on chromosome 4q13.2 overlapping with *UGT2B15* and *UGT2B17* (encoding Uridine diphospho-glucuronosyltransferases) in 1,564 individuals (52%). The CNVs overlapping *UGT2B17* in most Thai people were found to be homozygous deletion (92.3%), similar to Japanese from Tokyo (JPT: 78.8%), Chinese from Beijing (CHB: 75.0%), and Chinese from Denver (CHD: 73.8%). The proportion of people containing homozygous deletion of *UGT2B17* was lowest in African population, with a frequency of 9.8% in Yoruban in Ibadan, Nigeria (YRI) and 9.5% in population with African ancestry in Southwest USA (ASW) (Table 4).

**Table 3:** Common CNVRs with at least 5% allele frequency in Thai population and their frequencies across HapMap3 populations.

ID	Chr	Start	Stop	Genes	THAI	CHB	CHD	JPT	ASW	LWK	MKK	YRI	GIH	MEX	TSI	CEU
1	1	187013019	187847262		0.06	0.04	0.05	0.06	0.00	0.00	0.00	0.00	0.00	0.00	0.00	0.00
2	2	34554235	35281044		0.06	0.00	0.00	0.00	0.00	0.00	0.00	0.00	0.00	0.00	0.00	0.00
3	3	163995351	164108689		0.52	1.00	0.95	0.98	0.66	0.86	0.89	0.78	0.42	0.40	0.56	0.49
4	3	163690547	163719579		0.17	0.37	0.28	0.36	0.00	0.00	0.00	0.00	0.00	0.00	0.01	0.00
5	3	65163493	65190844		0.06	0.05	0.07	0.13	0.00	0.00	0.01	0.00	0.09	0.14	0.06	0.13
6	3	116125098	116154405	ZBTB20	0.05	0.02	0.01	0.03	0.43	0.59	0.32	0.65	0.23	0.04	0.01	0.02
7	3	53001754	53021256	SFMBT1	0.05	0.14	0.11	0.05	0.06	0.00	0.00	0.00	0.06	0.30	0.18	0.16
8	4	69045672	69258302	TMPRSS11E2, TMPRSS11E, UGT2B17, UGT2B15	0.52	0.95	0.99	0.99	0.45	0.63	0.68	0.36	0.80	0.56	0.58	0.57
9	4	63352170	63377531		0.08	0.64	0.72	0.71	0.21	0.18	0.09	0.13	0.56	0.36	0.26	0.34
10	4	64328367	64483913		0.08	0.00	0.00	0.00	0.00	0.00	0.00	0.00	0.00	0.00	0.00	0.00
11	8	39350791	39509376		0.20	0.24	0.24	0.26	0.15	0.14	0.42	0.14	0.67	0.72	0.68	0.72
12	8	15444945	15580087	TUSC3	0.08	0.12	0.08	0.08	0.00	0.02	0.13	0.00	0.06	0.00	0.06	0.07
13	8	115595696	115932676		0.07	0.27	0.24	0.53	0.21	0.30	0.32	0.27	0.25	0.40	0.31	0.22
14	11	81181640	81203793		0.06	0.00	0.00	0.00	0.00	0.00	0.00	0.00	0.00	0.00	0.00	0.00
15	13	56604813	56850408	FLJ40296	0.10	0.39	0.32	0.41	0.87	0.91	0.81	0.91	0.07	0.22	0.17	0.21
16	14	40671757	40744653		0.14	0.24	0.32	0.28	0.04	0.02	0.04	0.00	0.19	0.50	0.30	0.38
17	14	105069589	105997070		0.10	0.00	0.00	0.00	0.00	0.00	0.00	0.00	0.00	0.00	0.00	0.00
18	14	42420358	44320168	FSCB	0.07	0.17	0.13	0.09	0.00	0.00	0.00	0.00	0.00	0.10	0.01	0.00
19	15	18294933	22368232	A26B1, CYFIP1, GOLGA8E, LOC283755, LOC283767, MAGEL2, MKRN3, NDN, NIPA1, NIPA2, OR4M2, OR4N4, TUBGCP5	0.12	0.20	0.11	0.10	0.11	0.08	0.08	0.11	0.24	0.22	0.06	0.16
20	15	32459510	32626301	GOLGA8A, GOLGA8B	0.07	0.00	0.00	0.00	0.00	0.00	0.00	0.00	0.00	0.00	0.00	0.00
21	16	31909325	33867424	LOC729355, LOC729355	0.20	0.75	0.79	0.71	0.36	0.38	0.29	0.31	0.50	0.90	0.75	0.67
22	17	41519743	42137359	ARL17, KIAA1267, LRRC37A2, LRRC37A, NSF	0.22	0.74	0.71	0.70	0.70	0.51	0.63	0.56	0.84	0.78	0.91	0.91
23	17	14030694	15533487	CDRT15, CDRT1, CDRT4, COX10, FAM18B2, FLJ45831, HS3ST3B1, PMP22, TEKT3, TRIM16	0.07	0.36	0.38	0.31	0.00	0.00	0.00	0.00	0.02	0.02	0.02	0.00
24	18	64862553	64919011	CCDC102B	0.24	0.25	0.26	0.43	0.00	0.01	0.05	0.00	0.02	0.06	0.05	0.05
25	19	20385941	20559157	ZNF826	0.11	0.21	0.18	0.23	0.17	0.11	0.24	0.19	0.19	0.10	0.15	0.09

CNVRs covering HLA, immunoglobulin superfamily, and OR genes were excluded due to their multiallelic nature, which might result in inaccuracy of CNV calling in these regions.

**Table 4:** Population frequency of CNVs overlapping with *UGT2B17* and proportion of the total CNVs that were homozygous deletion.

	ASW	YRI	MEX	LWK	TSI	CEU	MKK	GIH	CHD	CHB	JPT	THAI
Total population frequency	0.447	0.363	0.560	0.633	0.580	0.570	0.676	0.795	0.988	0.952	0.988	0.522
Homozygous deletion frequency	0.042	0.036	0.100	0.122	0.125	0.158	0.225	0.341	0.729	0.714	0.779	0.482
Homozygous deletion proportion	0.095	0.098	0.179	0.193	0.216	0.277	0.333	0.429	0.738	0.750	0.788	0.923

**Figure 3:**
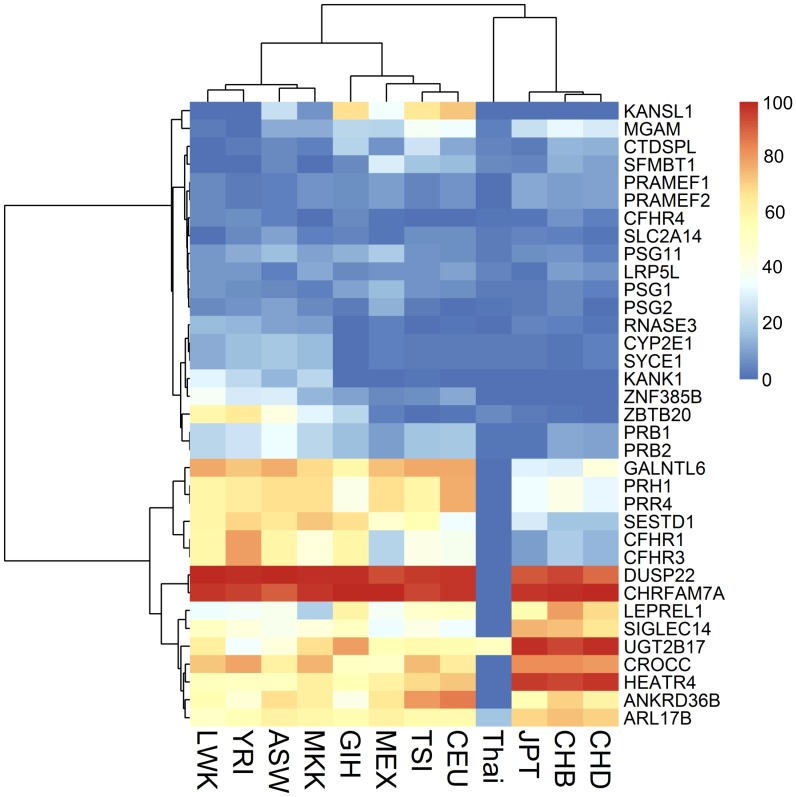
Hierarchical clustering analysis (HCA) of the 35 genes overlapping CNVs with statistically significantly different allele frequencies across HapMap3 populations as compared with Thais (permutation P-value <0.0002). The color bar on the right shows the color codes assigned to each frequency range in percent.

In an attempt to identify additional CNVs overlapping with genes that might be either more common or less common in the Thai population compared to HapMap3, the frequencies of CNVs between the Thai population versus each of the 11 HapMap3 populations were compared using a test of association. When a cut-off p-value of <0.01 was used, a total of 173 genes overlapping with CNVs were identified (Table S3 in File S1). To uncover the candidate genes representing Thai-specific CNVs, the top 20 genes showing the greatest difference in frequency between each population pair were chosen, which resulted in a non-redundant list of 35 genes (Table S4 in File S1; p<0.0002). Hierarchical clustering analysis (HCA) was performed on the scaled frequency data of these gene-overlapping CNVs to group the most similar population together based on frequencies. Although these CNVs were picked to highlight the difference between Thai and HapMap3 populations, the populations that showed the closest relationship to Thais were JPT, CHB, and CHD (Figure 3). Based on the HCA results, Asian populations were still the most similar to each other. Populations with European and African ancestries from HapMap3 samples were placed in a different clade from Asian populations. Hence, cautions should be taken when interpreting CNVs found in the Thai population using CNV databases created based on subjects with European or African ancestry.

### The Thai CNV database

**Figure 4:**
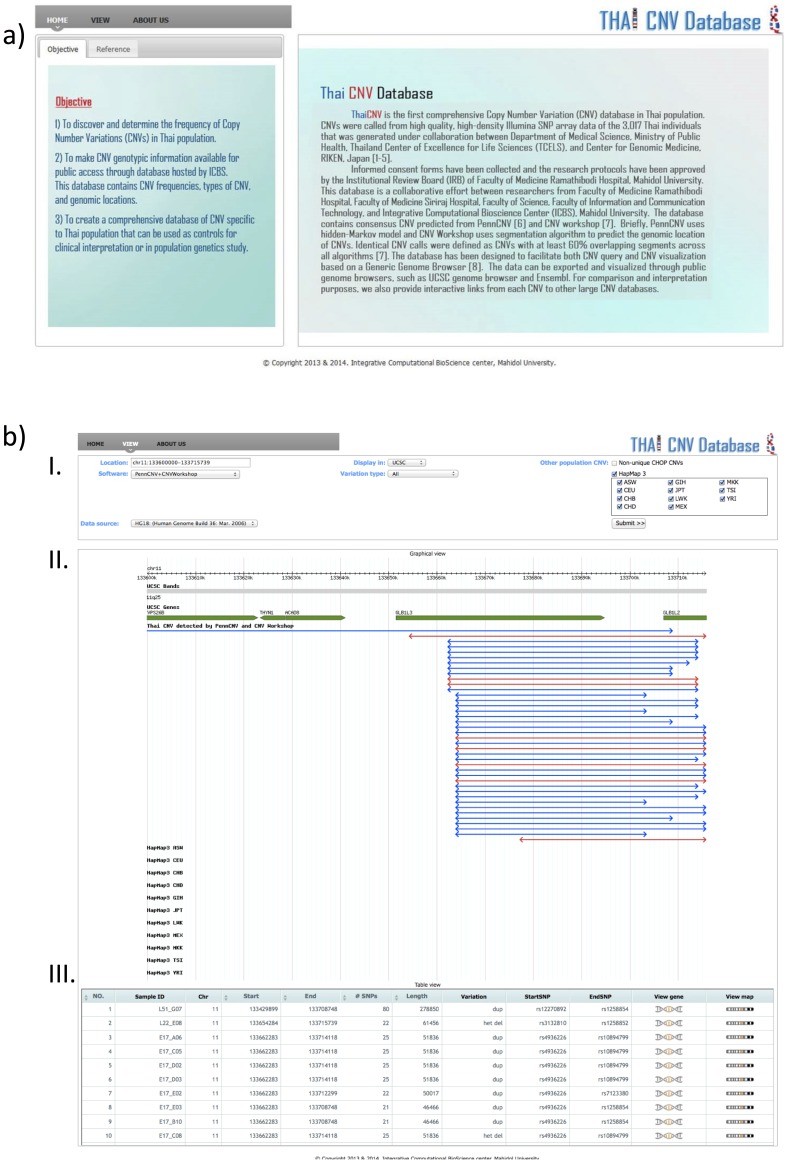
Thai CNV database. a) A screen-captured image of Thai CNV homepage (http://thaicnv.icbs.mahidol.ac.th/thaicnv/); b) An example of CNV search page. Red and blue lines indicate deletion and duplication CNVs, respectively. Arrowheads indicate the starting and ending genomic locations. Panel I - input panel; panel II - graphical view; panel III - table view.

As a significant fraction of CNVs were population specific, a web-based database containing CNVs identified in the Thai population was created to facilitate the clinical use of CNV in genetic diagnosis. The website is freely available, and can be accessed at http://thaicnv.icbs.mahidol.ac.th/thaicnv (Figure 4a). MySQL database schema is shown in Figure S1. The website allows users to query specific CNVs using either a genomic coordinate based on UCSC genome build hg18 or hg19. A list of CNVs identified in the Thai samples was listed in a table format, and a graphical interface of these CNVs was provided (Figure 4b). Links to a list of RefSeq genes overlapping with each CNV, and frequently used genome browsers namely UCSC genome browser, Ensembl, DGV, DECIPHER, and NCBI dbVar can be browsed directly from the list of CNVs shown in a table format. Users can also limit the type of CNVs, deletion or duplication to only be shown in the table. Furthermore, users can choose to see CNVs that were called by each CNV-calling algorithm or a combination of algorithms. Graphical interfaces of reference CNVs from HapMap3 and CHOP CNV (http://cnv.chop.edu/) were also provided for convenience. An example of unique CNVs in Thai population is shown in Figure 4b-II.

## Discussion

We have established a large reference CNV database for Thai population, which contains CNVs from 3,017 unrelated Thai individuals whose high-resolution Illumina SNP array-derived GWAS data were previously published. These subjects consisted of patients with infectious diseases namely tuberculosis, leprosy, and HIV/AIDS, patients with Thyrotoxic Hypokalemic Periodic Paralysis (THPP), and patients with Hb E/β-thalassemia. None of the subjects had other documented genetic disorders on top of their conditions at the time of diagnosis. Although Hb E/β-thalassemia is a genetic disease, it is a known single gene disorder with autosomal recessive inheritance resulting from compound heterozygous mutations in the *HBB* gene. Therefore, it may be assumed that CNVs in these Thai individuals are mostly benign, although the possibility that some of these CNVs might be associated with disease susceptibility cannot be completely ruled out. We also confirmed that only 3.27% of the Thai CNVs were overlapped with the known chromosomal disorders in DECIPHER v7.0. Our database is the most representative of the general Thai population to date, and is therefore suitable as a control for clinical interpretation of CNVs in Thai patients and related ethnic groups with potential genetic disorders.

By using rigorous filtering criteria as well as a combination of two different algorithms for CNV calling to avoid potential algorithm-specific errors [22], we identified a median of eight high confident CNVs per Thai individual. This number is considerably fewer than the medians of each HapMap3 population [12]. This may be because only autosomal CNVs were included in our study, whereas Hapmap3 used two denser SNP arrays, combining Illumina 1M with Affymetrix 6.0, and thus allowed a higher number of smaller CNVs to be detected with confidence (Table S5 in File S1). The estimated cumulative genome coverage of Thai CNVRs was 8.72%, which is similar to an earlier report using relatively homogeneous study population [8]. In accordance with HapMap3 study, the majority of Thai CNVs characterized were at low allele frequency, and the allele frequency spectrum of CNVs with >10% frequency was relatively similar between the Thai and HapMap3 populations. However, the larger Thai population sample size may contribute to the higher absolute number of low frequency CNVs in the Thai individuals observed.

To identify Thai-specific CNVRs, we examined the degree of match between the CNVRs characterized in the Thai and HapMap3 subjects, and found that approximately 80% of Thai CNVRs did not overlap with those of HapMap3. These CNVRs tended to be significantly smaller with a mean allele frequency of only 0.26%. The high amount of rare Thai-specific CNVRs (<0.5% frequency) may be explained, at least in part, by the fact that there is no Thai individual included in the multiracial HapMap3 study population. On the contrary, common Thai CNVRs (>5% frequency) showed a higher degree of match with HapMap3 CNVRs, reflecting that common CNVRs are shared regardless of ethnicity. These findings are in agreement with a previous study showing comparison between Korean CNVRs and CNVRs derived from a public CNV depository database, DGV [8].

Furthermore, we determined a set of gene-overlapping CNVs, of which frequencies were statistically significantly different between Thais and each HapMap3 population. Uridine diphospho-glucuronosyltransferase 2B17 (*UGT2B17*) *was* among the top 35 genes, and it was also overlapped with the most common Thai CNVRs. *UGT2B17* is the most active enzyme in glucuronidation of androgens, which is a major source for estrogen. Both androgen and estrogen help stimulate bone formation in humans. Higher *UGT2B17* gene copy number (≥ one copy) is associated with increased risk of osteoporotic hip fracture in Chinese and Caucasian populations, while homozygous deletion of *UGT2B17* is a protective factor [23]. Hip fracture rates after age adjustment are more common in Scandinavian and North America than in Southern Europe, Asia, and Latin America [24]. Interestingly, our data correspondingly demonstrated a higher number of East Asian populations (CHB, CHD, JPT) with *UGT2B17* homozygous deletion than Caucasian populations (CEU, TSI). The frequency difference of *UGT2B17* homozygous deletion across populations, therefore, is consistent with the lower risk of osteoporotic hip fracture found in Asian as compared to Caucasian populations. The number of Thais with *UGT2B17* homozygous deletion falls between that of East Asians and Caucasians. However, a large molecular epidemiological study is needed to clarify the incidence and prevalence of osteoporotic fracture of the hip in Thai population and establish the correlation between *UGT2B17* copy number variation and osteoporosis risk.

It is known that there is a subtle genetic difference within the Asian populations that may render genetic information not completely interchangeable [25]. A study has shown that although the similarity in allele frequency and linkage disequilibrium between Thais and East Asians is high, but at least 5% of drug-related alleles in Thais are not captured by East Asian-derived haplotype-tagging SNPs [26]. In line with the above observation, a hierarchical clustering analysis using allele frequencies of CNVs containing the 35 top candidate genes across populations could successfully separate the 12 study populations into three groups according to their ancestral origin; Africans (LWK, YRI, ASW, MKK), Europeans (GIH, MEX, TSI, CEU), Asians (Thai, JPT, CHB, CPT). Based on the CNV occurrences, Thais were clustered near the East Asian populations, yet, were clearly distinguishable from them. Hence, cautions should be taken when interpreting uncertain clinical significance CNVs found in the Thai population using reference CNV databases created based on other non-Thai populations. Lack of reference CNVs available in Caucasian-populated CNV databases at Thai-specific CNV locations might lead to misinterpretation of Thai CNVs from uncertain significance to pathogenic.

In summary, we have established a reference CNV database for Thais, which is the largest of its kind to date. This database will serve as a valuable resource of reference CNVs for clinical diagnosis of Thai patients with genetic disorders, and to identify Thai-specific novel CNVs and CNVRs that were differentially distributed among other populations. From this study, we have characterized population-specific CNVs supporting the notion that a population-specific CNV database will greatly contribute to more accurate interpretation of clinical significance of CNVs.

## Supporting Information

Figure S1 MySQL schema for Thai CNV database.
(TIFF)

File S1 **Supporting Tables S1–S5.** Table S1: Sample size of all populations used in this study. Table S2: Characteristics of CNVs discovered by PennCNV (HMM) and CNV Workshop (CBS). Table S3: List of 173 genes overlapping CNVs with statistically significantly different allele frequencies across HapMap3 populations as compared with Thais (p-value <0.01). Table S4: The 35 genes overlapping CNVs with statistically significantly different allele frequencies across HapMap3 populations as compared with Thais (p-value <0.0002); the pink, blue, and yellow cells represent homozygous or heterozygous deletion CNVs, duplication CNVs, and complex CNVs, respectively. Table S5: Summary of genotyping platforms and CNV-calling algorithms used in this study.
(XLS)
