# Supplementary figures and images for: Copy Number Variation in Thai Population
Source: PLoS One. 2014 Aug 13;9(8):e104355. doi: 10.1371/journal.pone.0104355 (PMC4131886; doi:10.1371/journal.pone.0104355)

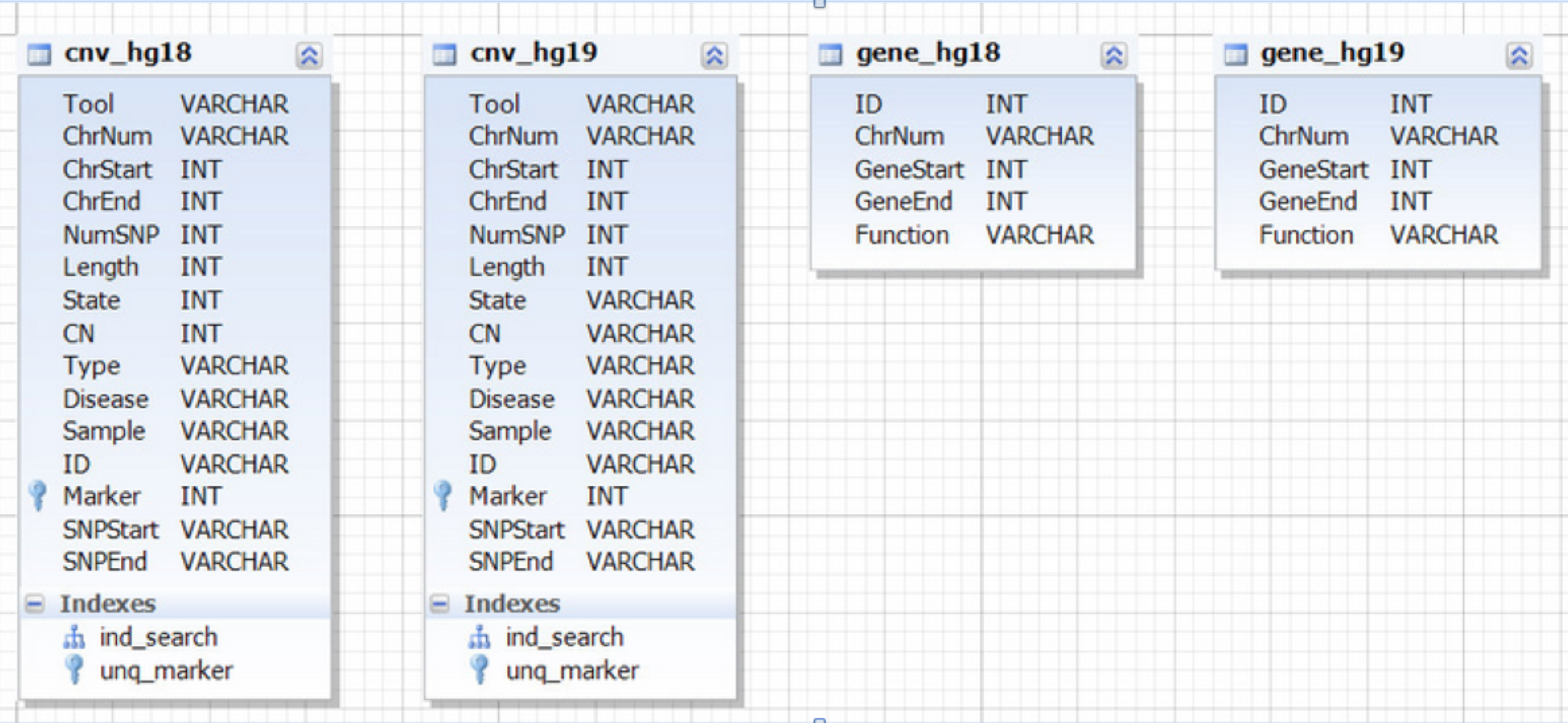

Supplement: Figure S1 — MySQL schema for Thai CNV database. (TIFF) [file pone.0104355.s001.tiff]
